# Development and Application of Fruit Color-Related Expressed Sequence Tag-Simple Sequence Repeat Markers in *Abelmoschus esculentus* on the Basis of Transcriptome Sequencing

**DOI:** 10.3389/fpls.2022.907895

**Published:** 2022-05-23

**Authors:** Xia An, Xiahong Luo, Tingting Liu, Wenlue Li, Lina Zou

**Affiliations:** Zhejiang Xiaoshan Institute of Cotton & Bast Fiber Crops, Zhejiang Institute of Landscape Plants and Flowers, Zhejiang Academy of Agricultural Sciences, Hangzhou, China

**Keywords:** *Abelmoschus esculentus*, transcriptome sequencing, fruit color, genetic diversity, EST-SSR, markers

## Abstract

*Abelmoschus esculentus* is a medicinal and edible plant that contains large amounts of active ingredients, including anthocyanins, polysaccharides, flavonoids, and terpenoids. However, because of a relative lack of molecular research, there are few molecular markers applicable for this plant species. In this study, on the basis of *A. esculentus* fruit color-related transcriptome sequencing data, we analyzed the patterns of simple sequence repeats (SSRs) in differentially expressed genes (DEGs) and revealed the biological processes and metabolic pathways associated with the related genes. We also designed primers for SSR loci to develop SSR molecular markers. Primers were synthesized using a DEG associated with a protein–protein interaction network. Polymorphic SSR markers were screened for the subsequent examination of *A. esculentus* germplasm resources and fruit color association analysis. The results indicated that 24.98% of the unigenes contained SSR motifs. Single-base (mononucleotide) repeats were the main SSRs, followed by trinucleotide and dinucleotide repeats. We selected 47 expressed sequence tag (EST)-SSR primer pairs for the genotyping of 153 *A. esculentus* varieties/lines. We ultimately obtained 21 EST-SSR markers suitable for genotyping. A generalized linear model-based association analysis detected two EST-SSR markers significantly associated with *A. esculentus* fruit color. In conclusion, several EST-SSR and SSR molecular markers in *A. esculentus* were developed in this study. The fruit color-associated markers may be useful for the molecular marker-assisted breeding of new *A. esculentus* varieties.

## Introduction

*Abelmoschus esculentus* (okra) is an important cash crop with a high nutritional value (Adelakun et al., 2009). Polysaccharides in *A. esculentus* have anti-depressant and anti-inflammatory effects and its mucilage is rich in functionally active and antioxidant substances (Tian et al., 2015). Additionally, *A. esculentus* is an important medicinal and edible plant with beneficial effects on human health (Liao et al., 2012). However, biotechnology-based methods for accelerating the genetic improvement of *A. esculentus* have seldomly been reported and there are few related molecular markers (Mishra et al., 2017). Mainstream molecular markers, including simple sequence repeats (SSRs) and inter-simple sequence repeats (ISSRs) (Yuan et al., 2014); have been used for the genetic analysis and molecular marker-assisted breeding of *A. esculentus* (Yildiz et al., 2015). As transcriptome sequencing has become more affordable, transcriptome sequencing-based SSR molecular markers have been increasingly developed for many species, including *A. esculentus*. Some transcriptome sequencing-based expressed sequence tag (EST)-SSR molecular markers for *A. esculentus* has also been developed (Schafleitner et al., 2013).

The number of available molecular markers is much lower for *A. esculentus* than for other crops (Yuan et al., 2014; Yildiz et al., 2015). Among the transcriptome-based molecular markers, EST-SSRs developed using unigenes have specific tendencies because of the spatiotemporal specificity of gene expression. Thus, they are useful for the genetic improvement of *A. esculentus*.

*Abelmoschus esculentus* is a medicinal and edible vegetable, and its edible part is mainly the tender pod. There is considerable diversity in *A. esculentus* fruit colors (e.g., light green, green, dark green, pink, and mauve). Studies have shown that anthocyanins can protect against radiation, while also delaying aging and decreasing blood lipid levels (Chao et al., 2014; Fan et al., 2014). They also have some inhibitory effects on cancer (Wang and Stoner, 2008). Anthocyanin biosynthesis is a secondary metabolism-related process that has been extensively studied in various plants, including *Arabidopsis thaliana* (Matsui et al., 2008), maize (Selinger and Chandler, 1999), and other model plants, but there has been limited related research on *A. esculentus*. The diversity in fruit colors is usually associated with differences in the abundance of anthocyanins, which can scavenge reactive oxygen species through redox reactions and contribute to plant stress resistance and anti-aging health effects in humans. Fruit color is regulated by both genes and environmental conditions. Molecular markers related to fruit color may be used to screen for desirable fruit colors at the seedling stage, thereby accelerating the genetic improvement of *A. esculentus*. In this study, we developed transcriptome sequencing-based EST-SSR markers related to *A. esculentus* fruit color, providing okra researchers with additional molecular markers. Moreover, a set of differentially expressed genes (DEGs) and the associated protein–protein interaction (PPI) networks were analyzed in this study. Specific primers were selected and then validated using 153 *A. esculentus* germplasm materials. The polymorphism of the newly developed EST-SSR molecular markers and their utility for analyzing the genetic diversity of germplasm resources and population structures were investigated. Finally, an association analysis was performed for *A. esculentus* fruit color. In this study, we generated a new set of EST-SSR molecular markers that may be exploited for the molecular breeding of *A. esculentus*.

## Materials and methods

### Materials

A total of 153 *A. esculentus* germplasm resources (Supplementary Table 1) collected worldwide were used for screening and validating EST-SSRs and conducting a marker–fruit color association analysis.

### *De novo* Sequencing and Development of SSR Markers

The pink (No. AE70) and dark green (No. AE4) capsules of *A. esculentus* were collected, immediately frozen in liquid nitrogen, and stored at −80°C for the following experiments. Total RNA was extracted from the frozen samples using the TRIzol reagent (Invitrogen, CA, United States) as previously described (An et al., 2020). The RNA quality was checked by agarose gel electrophoresis as well as by an analysis using the Agilent 2100 Bioanalyzer (Agilent, CA, America) before further processing (An et al., 2020). The cDNA libraries were sequenced using the illumina novaseq 6000 platform. Transcripts were *de novo* assembled using the default parameters of Trinity and then further clustered into unigenes using the Corset software (Davidson and Oshlack, 2014). The assembled unigenes were imported into the MISA software for the SSR analysis. The type and frequency distribution of the SSR motifs were recorded. The repetitive motifs of SSRs were analyzed according to the following criteria: number of repeating mononucleotides ≥10; number of repeating dinucleotides ≥6; and number of repeating trinucleotides, tetranucleotides, pentanucleotides, and hexanucleotides ≥5. Specific SSR primers were designed on the basis of the unigene sequences using Primer3. The primers were designed to amplify fragments between 100 and 400 bp long.

### Gene Ontology and Kyoto Encyclopedia of Genes and Genomes Enrichment Analyses and PPI Network Analysis of DEGs

To investigate the biological functions of the SSR-containing unigenes and SSR-containing DEGs associated with PPI networks (e.g., the main biological processes, molecular biological functions, cellular components, and metabolic pathways), Gene Ontology (GO; Young et al., 2010), and Kyoto Encyclopedia of Genes and Genomes (KEGG; Mao et al., 2005) enrichment analyses were performed using TBTools (Wang et al., 2010).

String^1^ was used to analyze the PPI networks of DEGs, whereas Cytoscape was used to visualize the PPI networks.

### DNA Extraction

Approximately 200 mg young *A. esculentus* capsules were ground to a fine powder in liquid nitrogen. Total genomic DNA was extracted from the ground material using the DNAsecure Plant Genomic DNA Extraction kit (DP320-02; Tiangen, China). An approximately 2-μl aliquot of the DNA solution was collected to determine the DNA concentration and quality using the NanoDrop Microvolume UV Spectrophotometer (Model ND1000). A 4-μl aliquot of the DNA solution was analyzed by 1% agarose gel electrophoresis to check the DNA integrity. The DNA detected as a clear band was used for the subsequent SSR genotyping experiments.

### SSR Genotyping

Fluorescent SSR primers were synthesized by adding FAM to the 5′ end. These primers as well as Phi29 DNA Polymerase (TransGen; Cat. No. LP101-01) were used for a PCR amplification. The 20-μl reaction volume consisted of 1 μl each 2 μM primer, 2 μl DNA template, 2 μl buffer, 0.3 μl TransTaq, 1.6 μl dNTPs, and 12.1 μl ddH_2_O. The PCR program was as follows: 94°C for 4 min; 35 cycles of 94°C for 30 s, 56°C for 90 s, and 72°C for 1 min; 72°C for 5 min and then 4°C for storage. A 1-μl aliquot of the PCR product was analyzed using the ABI 3730xl capillary electrophoresis DNA analyzer, after which the GeneMapper 4.0 software was used to read and export the SSR genotyping data. More specifically, GeneMapper exported the size of the amplified fragment for each primer pair and converted it to a format suitable for the programs used for the subsequent analyses.

### Genetic Diversity Analysis

The SSR genotyping data were imported into the POPGENE (v1.32) software, which was used to calculate the number of alleles (Na), number of effective alleles (Ne), observed heterozygosity (Ho), expected heterozygosity (He), and Shannon diversity index (I) for each SSR marker in the diploid codominant mode. The PowerMarker (v3.25) software (Liu and Muse, 2005) was used to calculate the polymorphism information content (PIC) value. The genetic similarity (Jaccard coefficient) between two samples was calculated using the NTSYSPC (v2.10e) software (Adams and Rohlf, 2000).

### Genetic Structure Analysis

Nei and Takezaki (1983) genetic distance determined on the basis of the allele frequency was calculated using the PowerMarker (v3.25) software. A neighbor-joining phylogenetic tree was constructed using the MEGA (v7.0) software. The SSR genotyping data were imported into the Structure (v2.0) software. The *K* value was set as 1–20, with three replicates. The Markov chain Monte Carlo method was used, with 100,000 iterations and a burn-in of 10,000 iterations. The Structure (v2.0) results were analyzed using the Structure Harvester online tool (Earl and vonHoldt, 2011). The curve of the change in Δ*K* with *K* was plotted to determine the best *K* value. Then, the ind files corresponding to the three replicated runs using the best K value were downloaded and imported into the Clumpp (v2.0) software, which merged the three results into a Q value matrix (Jakobsson and Rosenberg, 2007). The Q plot was created using Structure (v2.0). Finally, the results of the neighbor-joining cluster and Structure analyses were compared to analyze the population genetic structure.

### Examination of the Okra Fruit Pod Color and Association Analysis

An analysis of *A. esculentus* fruit (about 9 cm long) revealed five distinct colors (light green, green, dark green, pink, and mauve). To generate phenotypic data for the EST-SSR marker–fruit color association analysis, the colors were assigned a value as follows: light green = 1, green = 2, dark green = 3, pink = 4, and mauve = 5. A preliminary association analysis was performed on the basis of the SSR genotyping results and the fruit color phenotypic data for 153 *A. esculentus* germplasm resources. The genotypic and phenotypic data were imported into the TASSEL 2.1 software and the loci with MAF < 0.05 were excluded. The association analysis was completed according to the generalized linear model (GLM). The threshold for determining that a molecular marker was significantly associated with *A. esculentus* fruit color was p_Marker <0.05. When the value was close to 0.05, the molecular marker was also considered to be associated with fruit color, but this association required further validation.

## Results

### Genomic SSR Analysis

We obtained 64,092 unigenes (total length of 68,344,383 bp) by splicing the transcriptome sequencing reads. The SSRs in the unigenes were analyzed using the MISA software (Table 1), which revealed 9,937 SSRs in 8,559 unigenes (i.e., 13.4% of all unigenes). On average, each sequence contained 1.16 SSRs. Additionally, 1,168 sequences had more than one SSR. Moreover, 473 SSRs had a compound formation. The unigenes containing SSRs included 372 fruit color-related DEGs, of which 178 and 194 had upregulated and downregulated expression levels, respectively.

**Table 1:** Overview of the unigenes containing simple sequence repeats.

Type	No.
Total number of sequences examined	64,092
Total size of examined sequences (bp)	68,344,383
Total number of identified SSRs	9,937
Number of SSR containing sequences	8,559
Number of sequences containing more than one SSR	1,168
Number of SSRs present in compound formation	473

**Figure 1:**
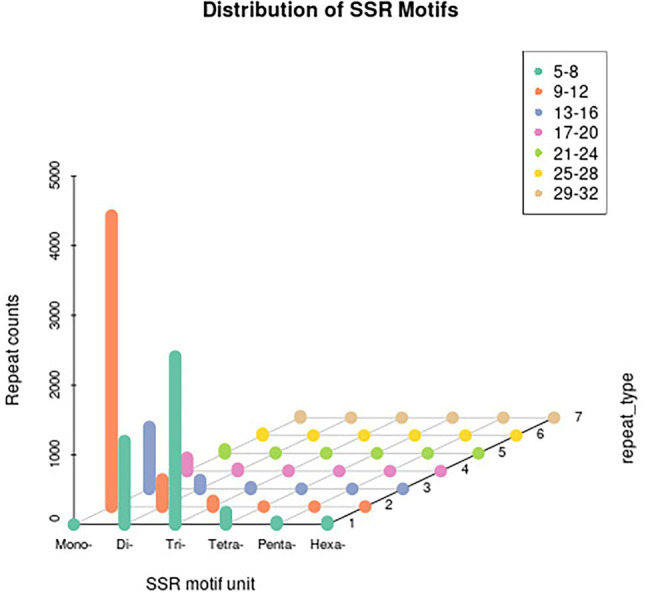
Distribution of SSR motifs in unigenes.

Among the detected SSRs, single-base repeats (mononucleotide motifs) accounted for the largest proportion, followed by trinucleotide motifs and dinucleotide motifs (Figure 1). We designed SSR primers for 7,675 unigenes (three primer pairs per SSR) and constructed a marker library for screening polymorphic EST-SSR markers (Supplementary Table 2).

To further characterize the unigenes containing SSRs and their functions, we performed GO and KEGG enrichment analyses (Supplementary Figures 1, 2). The main GO terms assigned to the unigenes containing SSRs were as follows: DNA-binding transcription factor activity, transmembrane transporter activity, and organic cyclic compound binding (molecular function category); transporter complex, ribonucleoprotein complex, and transcription regulator complex (cellular component category); biological regulation, cellular response to stimulus, and response to chemical (biological process category). The significantly enriched KEGG pathways among the unigenes containing SSRs were transcription factors (03000), plant hormone signal transduction (04075), and nicotinate and nicotinamide metabolism (00760).

### Protein–Protein Interaction Network Analysis of DEGs

**Figure 2:**
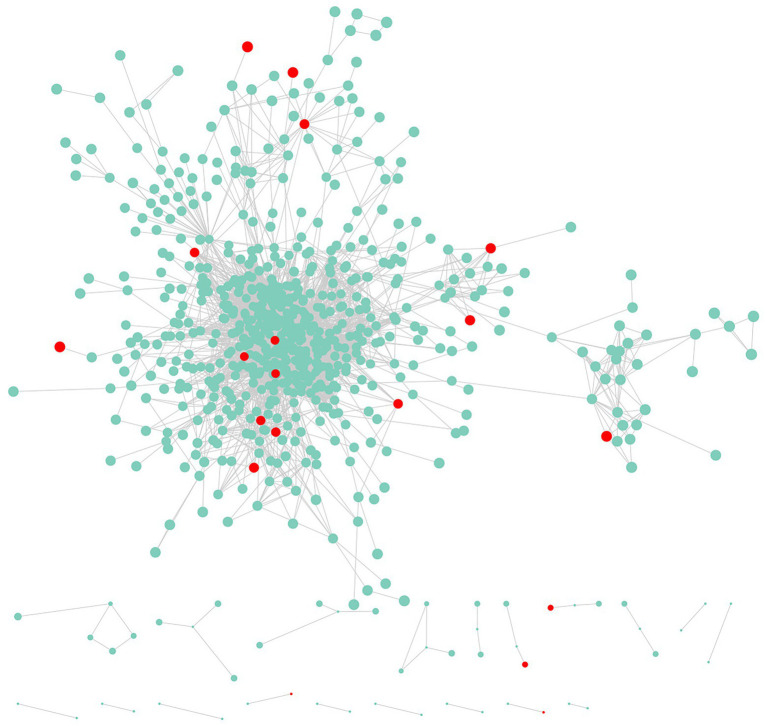
Genes and protein–protein interaction (PPI) networks associated with the newly developed EST-SSR markers.

**Figure 3:**
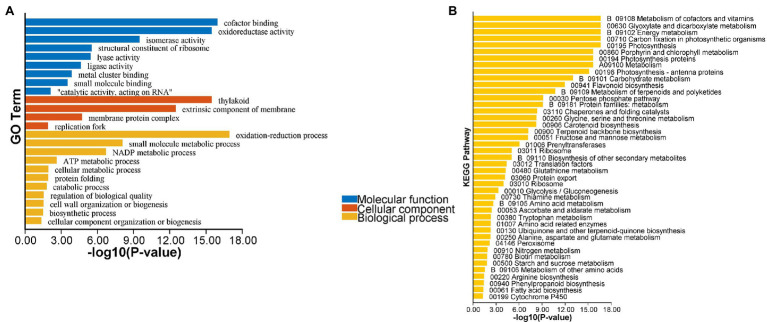
Enrichment analysis of the differentially expressed genes (DEGs) associated with PPI networks. **(A)** Gene Ontology (GO) enrichment analysis of the PPI network-associated DEGs; **(B)** Kyoto Encyclopedia of Genes and Genomes (KEGG) enrichment analysis of the PPI network-associated DEGs.

The transcriptome analysis revealed 2,186 DEGs, of which 957 DEGs (206 and 751 with upregulated and downregulated expression levels, respectively) may encode proteins involved in PPI networks (Figure 2). In total, 128 DEGs associated with PPI networks had at least one SSR that could be detected with the SSR primers designed in this study. The main GO terms assigned to the DEGs associated with PPI networks were as follows: oxidation–reduction process, small-molecule metabolic process, and NADP metabolic process (biological process category); cofactor binding, oxidoreductase activity, and isomerase activity (molecular function category; Figure 3A); thylakoid, extrinsic component of membrane, and membrane protein complex (cellular component category). Furthermore, the main enriched KEGG pathways were metabolism of cofactors and vitamins (B09108), glyoxylate and dicarboxylate metabolism (00630), and energy metabolism (B09102; Figure 3B).

### Validation of EST-SSR Molecular Markers

To validate the developed SSR markers and screen for polymorphic markers, we synthesized 47 primer pairs targeting the SSRs of the PPI-associated DEGs for an analysis of the genomic DNA extracted from 153 *A. esculentus* varieties/lines. Finally, 21 polymorphic EST-SSR markers were obtained, with a polymorphism rate of 44.7%. The genotyping data for the 21 polymorphic markers were used to analyze the genetic diversity of these markers and the genetic structure of *A. esculentus* germplasm resources.

### Single-Locus Genetic Diversity

We evaluated the genetic diversity of newly developed EST-SSR molecular markers in *A. esculentus* germplasm resources (Table 2). The analysis of 21 EST-SSR markers using 153 *A. esculentus* materials indicated the number of alleles was between 2 and 6 (average of 3.62). The number of effective alleles was between 1.01 and 2.04 (average of 1.22). The major allele frequency was between 0.50 and 0.99 (average of 0.90). The observed heterozygosity was between 0 and 0.99 (average of 0.16), whereas the expected heterozygosity was between 0.01 and 0.51 (average of 0.14). The PIC value was between 0.01 and 0.42 (average of 0.12). The Shannon diversity index was between 0.05 and 0.75 (average of 0.25). The genetic similarity coefficient for any two germplasm resources was between 0.58 and 1.00 (average of 0.86). The data suggested that these markers were insufficient for completely distinguishing the 153 germplasm materials. Hence, additional EST-SSR markers were required.

**Table 2:** Analysis of 21 new Expressed Sequence Tag-Simple Sequence Repeat (EST-SSR) markers in 153 *Abelmoschus esculentus* accessions.

Marker	Major aallele frequency	Na	Ne	Obs_Het	Exp_Het	PIC	I
OREST1	0.98	4	1.05	0.02	0.05	0.05	0.14
OREST2	0.97	5	1.06	0.03	0.05	0.05	0.16
OREST4	0.50	3	2.03	0.99	0.51	0.38	0.73
OREST5	0.95	3	1.12	0.09	0.10	0.10	0.24
OREST9	0.70	6	1.77	0.21	0.44	0.37	0.75
OREST12	0.97	5	1.07	0.04	0.06	0.06	0.18
OREST13	0.99	3	1.02	0.01	0.02	0.02	0.06
OREST17	0.99	2	1.02	0.02	0.02	0.02	0.06
OREST18	0.92	3	1.17	0.09	0.14	0.13	0.29
OREST19	0.99	3	1.01	0.01	0.01	0.01	0.05
OREST20	0.97	2	1.07	0.00	0.07	0.06	0.15
OREST21	0.99	4	1.02	0.02	0.02	0.02	0.07
OREST22	0.99	3	1.03	0.03	0.03	0.03	0.08
OREST30	0.95	3	1.11	0.02	0.10	0.09	0.21
OREST32	0.99	2	1.02	0.02	0.02	0.02	0.06
OREST33	0.99	4	1.03	0.03	0.02	0.02	0.08
OREST36	0.91	4	1.20	0.19	0.17	0.16	0.34
OREST37	0.71	4	1.72	0.58	0.42	0.34	0.65
OREST38	0.99	4	1.03	0.03	0.03	0.03	0.08
OREST39	0.50	4	2.04	0.95	0.51	0.42	0.75
OREST41	0.97	5	1.05	0.05	0.05	0.05	0.15
Mean	0.90	3.62	1.22	0.16	0.14	0.12	0.25

### Genetic Structure Analysis

**Figure 4:**
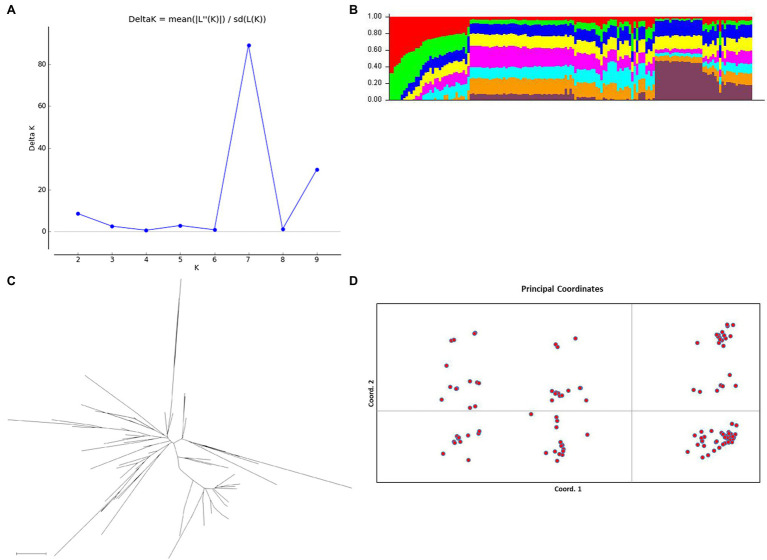
Analysis of the *Abelmoschus esculentus* germplasm resource population structure. **(A)** Change in Δ*K* with *K*. At *K* = 7, Δ*K* corresponded to the peak inflection point, implying the optimal number of populations was 7. **(B)** Q plot. The *y*-axis presents the *Q* values, which reflect the probability of dividing the corresponding material into a specific cluster, with different colors representing different clusters. **(C)** Neighbor-joining phylogenetic tree based on Nei’s genetic distance. **(D)** Principal coordinate analysis (PCoA) involving two-dimensional principal coordinates. Both PC1 and PC2 are principal components. The first three principal components explained 76.62% of the variation in the population structure.

The genotyping data for the 21 SSR loci were used for the analysis of the population genetic structure of 153 *A. esculentus* germplasm materials. An *a priori* model in the Structure software was used to infer the probability that each material was classified into a specific sub-population. The results were subsequently imported into Structure Harvester to analyze the change in ΔK with K (Figure 4A). When *K* = 7, Δ*K* corresponded to the inflection point. Thus, the number of optimal subpopulations in these 153 materials was 7. We combined the Q matrices of the three replicates corresponding to the optimal K value using Clumpp to generate the Q-Q plot (Figure 4B). According to the classification criteria, the samples with *Q* > 0.6 were clearly classified into specific clusters, whereas those with *Q* < 0.6 were classified into mixed clusters. The 153 materials were classified into seven clusters. With the exception of one material that was clearly classified into a specific cluster, the examined materials belonged to mixed clusters.

To further analyze the population genetic structure, PowerMarker was used to calculate Nei’s genetic distances according to the allele frequency. Additionally, the neighbor-joining method was used for a cluster analysis (Figure 4C) and to perform a two-dimensional principal coordinate analysis (PCoA; Figure 4D). The first three principal components (PC1, PC2, and PC3) explained 76.62% of the total variance (Figure 4D). The phylogenetic relationships among germplasm materials were assessed on the basis of the distances between the scattered points in the graph (Figure 4D). The results of the PCoA, the neighbor-joining cluster analysis, and the Structure-based cluster analysis revealed a lack of an obvious stratification among the *A. esculentus* germplasm resources. These results may be relevant for association analyses involving natural *A. esculentus* groups.

### Fruit Color–EST-SSR Association Analysis

**Figure 5:**
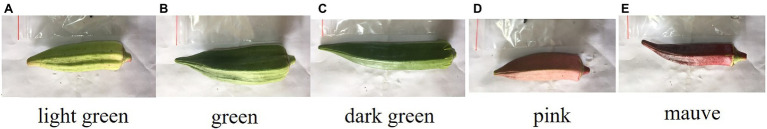
*Abelmoschus esculentus* fruit colors. **(A)**. light green, **(B)**. green, **(C)**. dark green, **(D)**. pink, and **(E)**. mauve.

**Figure 6:**
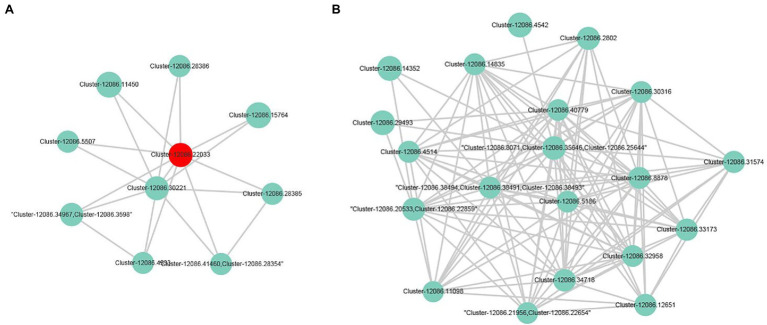
Sub-network containing the two fruit color-associated markers. **(A)** represents the gene in which the association marker OREST22 is located and the DEGs in which it is interassociated. **(B)** represents the DEGs of the gene containing the potential association marker OREST1 and its protein interaction.

In this study, 153 *A. esculentus* germplasm materials were classified into different groups according to their fruit colors (light green, green, dark green, pink, and mauve; Figure 5). After assigning values (1–5) to the colors, the phenotypic data were imported into the TASSEL 2.1 software along with the EST-SSR genotypic data for a GLM association analysis. Two EST-SSR markers were revealed to be associated with *A. esculentus* fruit color (Table 3). Marker OREST22 was significantly associated with fruit color (*p* = 0.0235) and explained 4.94% of the phenotypic variation. Additionally, OREST22 was located in unigene cluster-12086.22033, which was annotated as a sulfite reductase (ferredoxin) and a chloroplastic protein (*Herrania umbratica*). The expression of unigene cluster-12086.22033 was significantly downregulated according to the transcriptome data (Figure 6A). Marker OREST1 was also associated with fruit color (*p* = 0.0615), although not significantly. This marker explained 5.97% of the phenotypic variation. Marker OREST1 was located in unigene cluster-12086.35646, which was annotated as a hypothetical protein. The transcriptome data revealed the significantly downregulated expression of unigene cluster-12086.35646 (Figure 6B). These results suggested that the genes containing OREST1 or OREST22 might be functional genes related to *A. esculentus* fruit color. Moreover, the two EST-SSR markers may be applicable for the genetic improvement of *A. esculentus*.

**Table 3:** Two EST-SSR markers associated with *Abelmoschus esculentus* fruit color.

Locus	p_Marker	Rsq_Marker	Gene_id	NR GI	NR Description
OREST1	0.0615	5.97%	Cluster-12086.35646	763,778,394	Hypothetical protein B456_007G309800, partial (*Gossypium raimondii*)
OREST22	0.0235	4.94%	Cluster-12086.22033	1,204,894,708	Sulfite reductase (ferredoxin), chloroplastic (*Herrania umbratica*)

Rsq_Marker indicates the contribution to the phenotypic variation, whereas p_Marker indicates the significance of the association.

## Discussion

The application of transcriptome sequencing technology has resulted in a rapid increase in the number of EST and EST-SSR markers in many plants, including tiger lily (Sun et al., 2022), common buckwheat (Liu et al., 2022), vegetable soybean (Zhang et al., 2013), and *Chrysanthemum morifolium* Ramat (Fan et al., 2022). However, there have been relatively few reports describing EST-SSR molecular markers in *A. esculentus*. The molecular markers that have been developed for *A. esculentus* have primarily been SSR markers (Schafleitner et al., 2013) and sequence-related amplified polymorphism (SRAP) markers (Gulsen et al., 2007). In this study, *A. esculentus* fruit color-related SSR molecular markers were developed on the basis of transcriptome sequencing data. Additionally, the SSR characteristics of fruit color-related genes were analyzed. We performed an SSR analysis using the unigenes obtained from *A. esculentus* transcriptome sequencing data. In an earlier study, SSR, ISSR, and SRAP markers were used to elucidate the genetic diversity among six okra varieties (El-Afifi et al., 2018). The number of unigenes identified in this study was similar to the number of unigenes detected in an earlier investigation, but the SSR motif distribution characteristics and the number of unigenes containing SSRs differed (Schafleitner et al., 2013). This inconsistency may be ascribed to the diversity in the expressed genes identified in different transcriptome sequencing experiments (Zhang et al., 2013).

The results of the sequence enrichment analysis of the SSR-containing unigenes implied these unigenes might be mainly associated with plant signaling pathways. Accordingly, the EST-SSR primers developed in this study may primarily anneal to plant signaling-related genes. The EST-SSR markers described herein may be used to assess the genetic diversity of an *A. esculentus* population as well as the diversity in specific genes/related traits among *A. esculentu*s germplasm resources (El-Afifi et al., 2018). The data generated in the current study may form the basis of future research conducted to further clarify the genetic diversity of *A. esculentu*s and to screen for breeding materials (Yuan et al., 2014).

We analyzed DEGs associated with PPI networks because groups of related genes often have similar functions and represent a large proportion of DEGs (Cao et al., 2021). An enrichment analysis revealed that the genes encoding proteins belonging to PPI networks might contribute to energy metabolism and photosynthesis (Cevallos-Casals and Cisneros-Zevallos, 2004). These genes might be important for fruit color formation. In the current study, we screened the SSR markers in these DEGs and then validated 47 EST-SSR molecular markers with trinucleotide repeats. Finally, 21 polymorphic EST-SSR markers with a high detection rate were obtained and used for the evaluation and association analysis of *A. esculentus* germplasm resources.

The PIC value can be used to estimate the utility of a marker for discriminating between genotypes (Nagl et al., 2011). Moreover, PIC values were previously used to assess the genetic diversity in okra (Yuan et al., 2014). The overall mean PIC value for the 21 EST-SSR markers developed in this study was lower than that of previously reported markers (Schafleitner et al., 2013; Yuan et al., 2014), but was within the PIC value distribution range determined in another study (Yildiz et al., 2015). The same trend was observed for the Shannon diversity index. On the basis of the heterozygosity, diversity index, and PIC value, three EST markers useful for the subsequent evaluation of *A. esculentus* germplasm resources were identified. We analyzed the polymorphism of 21 EST-SSR markers in 153 *A. esculentus* germplasm resources. Although these markers were significantly polymorphic, they could not distinguish all 153 materials. This may be interpreted as follows. First, the number of EST-SSR markers included in our analysis was insufficient for the number of examined materials. Second, EST-SSR molecular markers are located in exonic regions and their polymorphism is lower than that of SSRs in spacer regions.

Among the SSRs detected by the transcriptome sequencing analysis in this study, the single-base repeats were the most common, followed by the trinucleotide repeats. This is inconsistent with the findings of an earlier investigation by Schafleitner et al. (2013), in which trinucleotide and hexanucleotide repeats were detected as the predominant SSRs. This difference between studies may be explained by the diversity in gene expression, which leads to transcriptome bias (Schafleitner et al., 2013). Therefore, we selected trinucleotide SSR loci for the subsequent analysis (Jakobsson and Rosenberg, 2007). Clarifying genetic diversity and population structure is essential for elucidating the breeding history and genetic relationships of crops. The genetic structure of *A. esculentus* germplasm resources revealed a stratification phenomenon. However, obvious links among the populations indicated that the stratification phenomenon was insignificant. This genetic structure was in accordance with the fruit color-based classification of the germplasm resources. Although we detected five *A. esculentus* fruit color types, there were some intermediate types among the examined germplasm resources. Hence, the association between the marker patterns and phenotypic patterns may lead to a stratified structure.

We also conducted an association analysis of *A. esculentus* fruit color and detected one significantly associated marker as well as one potentially associated marker that remain to be experimentally verified. The significantly associated marker might be in a gene encoding a scavenger of reactive oxygen species, indicating that *A. esculentus* fruit color formation may be influenced by antioxidant activities (Chao et al., 2014; Fan et al., 2014). This possibility should be thoroughly investigated in future studies.

In conclusion, we developed EST-SSR markers for *A. esculentus* on the basis of fruit color-related transcriptome data and then selected SSR primers for the DEGs associated with PPI networks for screening and validation experiments. Finally, we obtained 21 new highly polymorphic EST-SSR markers applicable for genotyping. These markers may be used for analyzing the genetic diversity and population structure of *A. esculentus* germplasm resources. Furthermore, the molecular markers significantly associated with fruit color may be relevant for the genetic improvement of *A. esculentus* to produce novel varieties with desirable fruit colors.

## Data Availability Statement

The original contributions presented in the study are included in the article/Supplementary Material; further inquiries can be directed to the corresponding author. The raw transcriptome sequencing data presented in the study are deposited in the NCBI database repository, accession number SRR18741621 to SRR18741624.

## Author Contributions

XA: conceptualization, methodology, formal analysis, resources, data curation, writing–review and editing, visualization, supervision, project administration, and funding acquisition. WL: software. XA, WL, and TL: validation. XL: investigation. XA and LZ: writing–original draft preparation. All authors contributed to the article and approved the submitted version.

## Funding

This study was supported by the National Key R&D Program and Key Special Project of International Science and Technology Innovation Cooperation between Governments (2017YFE0195300), the Basic Public Welfare Research Program of Zhejiang Province (LGN20C150007), the National Natural Science Foundation of China (31801406), the China Agriculture Research System of MOF and MARA, China Agriculture Research System for Bast and Leaf Fiber Crops (CARS-16-S05), and the Water and Soil Health Discipline Integration Project of Zhejiang Academy of Agricultural Sciences.

## Conflict of Interest

The authors declare that the research was conducted in the absence of any commercial or financial relationships that could be construed as a potential conflict of interest.

## Publisher’s Note

All claims expressed in this article are solely those of the authors and do not necessarily represent those of their affiliated organizations, or those of the publisher, the editors and the reviewers. Any product that may be evaluated in this article, or claim that may be made by its manufacturer, is not guaranteed or endorsed by the publisher.

## Footnotes

^1^ https://string-db.org/

## Supplementary Material

Supplemental Figure 1 GO enrichment analyses of unigenes containing SSRs.

Supplemental Figure 2 KEGG enrichment analyses of unigenes containing SSRs.

Supplementary Table 2 Details regarding the EST-SSR primers used in this study.
