# Supplementary figures and images for: Development and Application of Fruit Color-Related Expressed Sequence Tag-Simple Sequence Repeat Markers in Abelmoschus esculentus on the Basis of Transcriptome Sequencing
Source: Front Plant Sci. 2022 May 23;13:907895. doi: 10.3389/fpls.2022.907895 (PMC9168766; doi:10.3389/fpls.2022.907895)

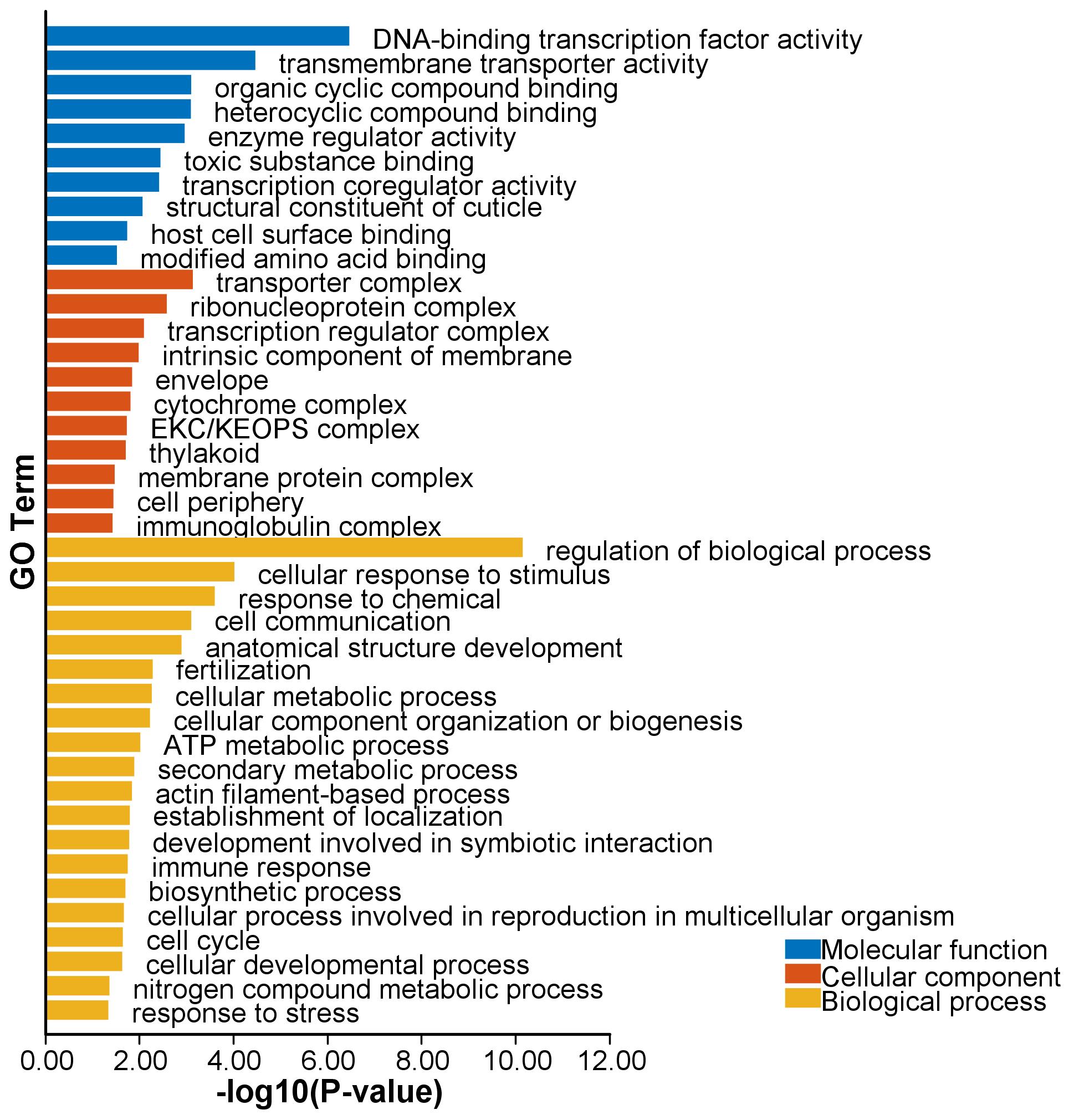

Supplement: Supplemental Figure 1 — GO enrichment analyses of unigenes containing SSRs. [file Image_1.JPEG]

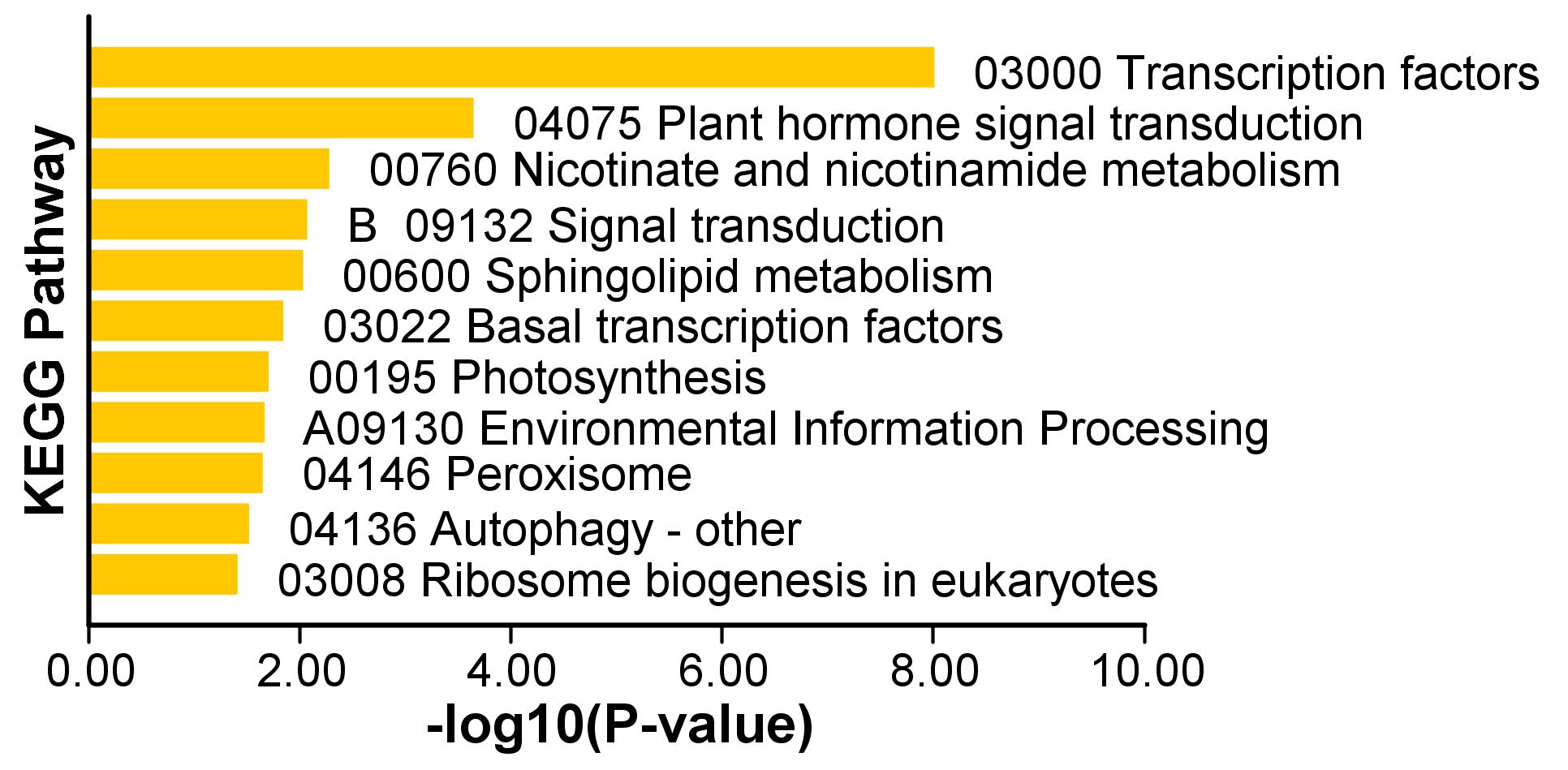

Supplement: Supplemental Figure 2 — KEGG enrichment analyses of unigenes containing SSRs. [file Image_2.JPEG]
